# Prestress-loading effect on the current–voltage characteristics of a piezoelectric p–n junction together with the corresponding mechanical tuning laws

**DOI:** 10.3762/bjnano.10.178

**Published:** 2019-09-06

**Authors:** Wanli Yang, Shuaiqi Fan, Yuxing Liang, Yuantai Hu

**Affiliations:** 1 Department of Mechanics, Hubei Key Laboratory of Engineering Structural Analysis and Safety Assessment, Huazhong University of Science and Technology, Wuhan 430074, China

**Keywords:** depletion layer, *I*–*V* characteristics, piezoelectric property, p–n junction

## Abstract

A model is proposed to study the diffusion of non-equilibrium minority carriers under the influence of a piezo potential and to calculate the corresponding current–voltage (*I*–*V*) characteristics of a piezoelectric p–n junction exposed to mechanical loading. An effective solution to describe this non-equilibrium process has been put forward including two concepts: the influence of prestress loading on p–n junctions in a quasi-electrostatic thermal equilibrium and the perturbation of small fields superposed on the obtained quasi-electrostatic solutions. A few useful results are obtained through this loaded p–n junction model. Under a forward-bias voltage, a tensile (compressive) loading raises (reduces) the potential barrier of the space charge zone (SCZ), i.e., produces an equivalent reverse- (forward-) electric voltage on the SCZ. When a piezoelectric p–n junction is exposed to a reverse-bias voltage, the current density monotonically decreases with increasing reverse voltage and gradually approaches saturation. A bigger tensile (compressive) loading produces a smaller (larger) saturation current density. The appearance of an equivalent voltage on the SCZ induced by prestress indicates that the performance of a p–n junction with the piezo effect can be effectively tuned and controlled by mechanical loadings. Meanwhile, numerical results show that a loading location closer to the SCZ produces a stronger effect on the *I*–*V* characteristics of a piezoelectric p–n junction, implying that the tuning effect of mechanical loadings depends on how much influence of the deformation-induced electric field can reach the SCZ. Furthermore, it is also found that the deformation-induced electric field becomes weak with increasing doping because the higher doping is corresponding to the stronger electric leakage. Thus, the higher mechanical tuning performance on higher doped piezoelectric p–n junctions requires the prestress loadings to be applied closer to the interface of p- and n-zone. This study on a non-equilibrium process of piezoelectric p–n junctions has significance for piezotronics.

## Introduction

With the new area of piezotronics proposed by Wang [1–2], researches on the fundamental characteristics of piezoelectric semiconductor structures and devices have been increasing. It should be emphasized that the most commonly utilized semiconductors at present are third-generation semiconductors, for instance, ZnO, GaN, CdS, and AlN, with wide bandgap, high breakdown electric field, high thermal conductivity, and even mechanical tunability [3]. They show numerous application prospects in electric devices and sensors, such as energy harvesters [4–13], MOSFETs [1,14], and acoustic charge transport devices [15–17]. For piezoelectric semiconductor devices, analyses on the static, time-harmonic and transient behaviors seem particularly important regarding their applications and development [18]. Zhang et al. [19] studied the static extensional behavior of a piezoelectric semiconductor nanofiber. Liang et al. [20] analyzed the fundamental characteristics of a cantilevered ZnO nanowire exposed to a transient end force. Recently, Fan et al. [21] and Zhang et al. [22] revisited the bending behavior of a cantilevered ZnO nanowire based on the linear phenomenological theory of piezoelectric semiconductors. In their studies, the electric leakage of a bent ZnO nanowire is connected to the semiconductor properties and concluded that a lower doping concentration is more suitable for a bent ZnO nanowire to harvest energy. Liang et al. [23] further studied the nonlinear effect of carrier drift on the performance of a ZnO nanogenerator following [21], and put forward a proper electrode configuration for the improvement of the nanogenerator performance. More recently, both time-harmonic and transient behavior have been investigated by Dai et al. [24] and Yang and co-workers [25]. The mechanical behavior of composite fibers with piezoelectric dielectrics and non-piezoelectric semiconductors have also been studied by Cheng et al. [26] and Luo and co-workers [27]. For piezoelectric p–n junctions, it becomes different from the above single-type doped semiconductor structures. A p–n junction usually consists of two differently doped semiconductors and has more complex fundamental characteristics. When a p–n junction exhibits piezoelectric properties, it becomes possible to tune and control the related fundamental characteristics of piezoelectric semiconductor devices by mechanical loading. A circular piezoelectric p–n junction was investigated by Luo et al. [28] based on a linear approximation for carrier fluctuations, in which the junction was exposed to anti-plane deformations. Fan et al. [29] studied the adjustability and controllability of a piezoelectric p–n junction by axial mechanical loadings and analyzed the influence of loading type and loading location on the fundamental characteristics of the junction. Semiconductor devices usually operate based on non-equilibrium carriers, and thus, the *I*–*V* characteristics of p–n junctions are especially important. There is a steady current through a p–n junction when an electric bias voltage is applied. Because mechanical loadings can tune electric potential and electric field of a piezoelectric p–n junction, the corresponding *I*–*V* characteristics can also be adjusted by applying strain or stress. Up to now, there is only a limited number of studies on tuning and controlling of non-equilibrium minority carriers (NEMC). Zhang et al. [14] studied the *I*–*V* characteristics of a p–n junction consisting of a p-type semiconductor without piezo-effect and an n-type piezoelectric semiconductor. Based on the hypothesis of depletion layer and the introduction of an undetermined parameter *w*_piezo_, they qualitatively obtained some new and interesting phenomena. Luo et al. [30] investigated the forward-bias *I*–*V* characteristics of a piezoelectric p–n junction under different mechanical loadings. They obtained linear *I*–*V* characteristics which they assumed to be a locally linearized approximation. We note that the recombination of non-equilibrium carriers was neglected in their study. Qin et al. [31] analyzed the electromechanical properties of a metal–GaN contact with different applied strains and bias voltages.

In this paper, a model is proposed to study diffusion of NEMC in a mechanically loaded piezoelectric p–n junction with applied bias voltage. In section “*I*–*V* characteristics of a mechanically loaded piezoelectric p–n junction”, the solving process for a low-level injection is divided into two steps: one is a quasi-electrostatic analysis of a mechanically loaded piezoelectric p–n junction under a bias voltage; and the other is to solve the continuity equation of NEMC by perturbation of small fields superposed on the obtained quasi-electrostatic solutions [32]. Distributions of electric potential, electric field and carrier concentration are quasi-electrostatically solved in section “Quasi-electrostatic analysis of a mechanically loaded piezoelectric p–n junction under a bias voltage” for a piezoelectric p–n junction exposed to mechanical loading and electric bias voltages. *I*–*V* characteristics are determined for a piezoelectric p–n junction under different mechanical loadings and with different bias voltages applied in section “Non-equilibrium analysis of a piezoelectric p–n junction under a bias voltage”. Numerical results show that a tuning can be achieved by mechanical loading. Finally, some general observations are summarized in section “Conclusion”.

## *I*–*V* characteristics of a mechanically loaded piezoelectric p–n junction

**Figure 1:**
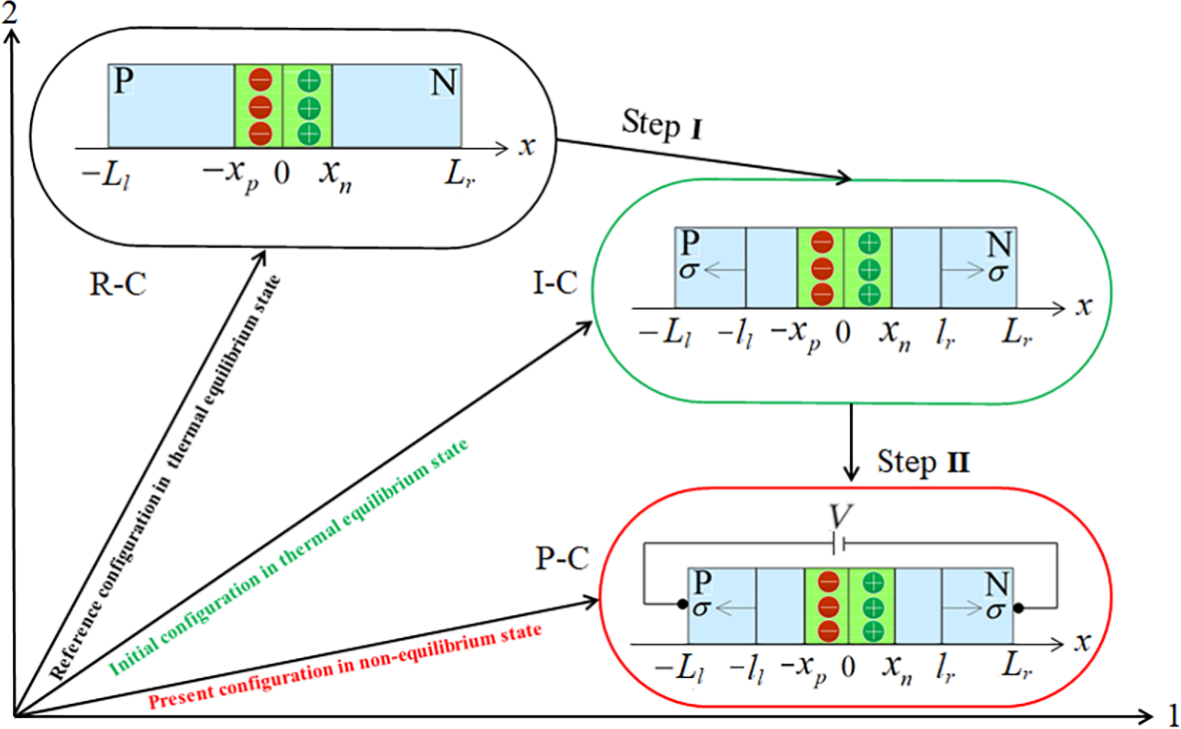
Three configurations of a piezoelectric p–n junction: 1) R-C stands for the piezoelectric p–n junction in the reference configuration without mechanical loadings and any electric bias voltage; 2) I-C stands for the p–n junction in the initial configuration exposed to mechanical loading without any electric bias voltage; 3) P-C stands for the p–n junction in the present configuration with mechanical loading and an electric bias voltage *V*.

In conventional semiconductor physics [33], the *I*–*V* characteristic of a common p–n junction under low-level injection is usually analyzed using a simple analogy based on the electrostatic analysis in thermal equilibrium, where the applied voltage (*V*) is assumed to drop in the depletion region such that its contact potential difference becomes 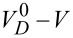 with 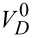 being the initial voltage. It implies that the electric potential remains almost unchanged in the two zones outside the depletion layer, the p-zone and the n-zone, and the electric field is almost vanishing there. Thus, the steady-state diffusion equation of minority carriers in the two zones has no terms related to the electric field. However, the situation becomes different for a piezoelectric p–n junction under mechanical loading. The deformation-induced electric field together with its derivative appears in the continuity equation of minority carriers and influences the redistribution of minority carriers in each zone outside the SCZ. In turn, the redistribution of minority carriers influences the electric field itself. Under low-level electric injection, the effect of minority carriers on the electric field is much smaller than the influence of deformation. Hence, the *I*–*V* characteristic of a mechanically loaded piezoelectric p–n junction under low-level injection can be obtained in through two steps (Figure 1). (I) Electric potential and electric field in both the p-zone and the n-zone induced by static loadings can be solved from the electrostatic analysis in thermal equilibrium; (II) the steady diffusion of minority carriers can be studied by using a perturbation technique of small fields superposed on the deformation-induced electric potential and electric field obtained in the first step.

In step I, we solve the distributions of electric potential field, electric field and carrier concentrations for a piezoelectric p–n junction in the I-C state shown in Figure 1. According to [29] the location of the loading has a great influence. In order to mechanically tune the performance of a piezoelectric p–n junction, the prestress should need to be applied close to the SCZ. A piezoelectric p–n junction can be divided into six parts schematically shown in the I-C state in Figure 1, where the *c*-axis of both p-zone and n-zone is set in the *x*-direction. Tensile/compressive stress, σ, is applied at *x* = −*l_l_* and *x* = *l_r_*. The coordinates −*x_p_* and *x_n_* are two boundaries of the SCZ. For −*l_l_* < *x* < *l_r_*, the electric displacement can be written as 
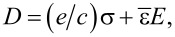
 where σ = *cS* – *eE*, 
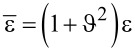
 and ϑ^2^ = *e*^2^/(*c*ε). In the above, strain *S* and electric field *E* can be expressed as *S* = d*u*/d*x* and *E* = −dϕ/d*x*, in which *u* and ϕ are displacement and electric potential, respectively. *c*, *e* and ε are elastic, piezoelectric and dielectric constant, respectively.

The one-dimensional Gauss’s law of a piezoelectric p–n junction can be written as

[1]
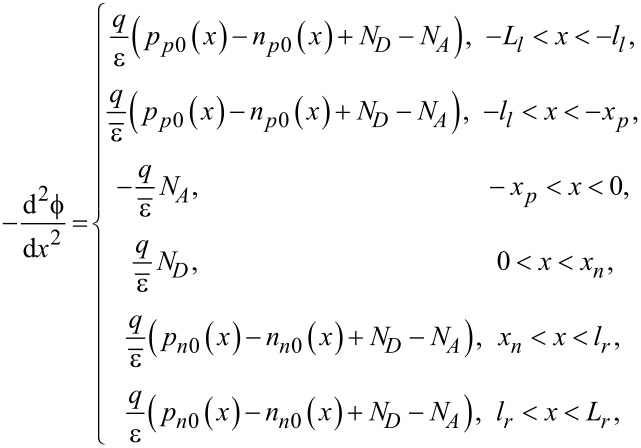


where (*p*_(_*_n_*_0,_*_p_*_0)_(*x*), *n*_(_*_n_*_0,_*_p_*_0)_(*x*)) and (*N_A_*, *N_D_*) are carrier concentrations and doping concentrations, respectively. The subscripts “*p*” and “*n*” represent p-zone and n-zone, respectively. *q* = 1.602 × 10^−19^ C is the electronic charge. For a piezoelectric p–n junction exposed to stress at *x* = −*l_l_* and *x* = *l_r_*, the carrier concentrations can be obtained from the vanishing current requirements in thermal equilibrium [29]

[2]
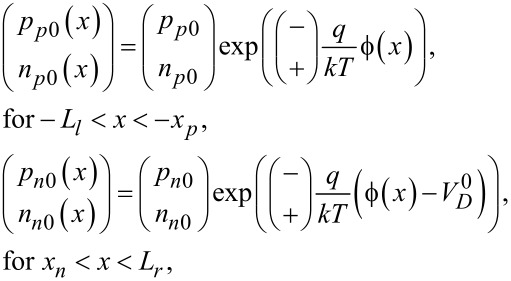


in which *k* is the Boltzmann constant, *T* represents temperature (as 300 K in our analysis) and 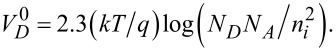 (*p_p_*_0_, *n_p_*_0_) and (*p_n_*_0_, *n_n_*_0_) are initial hole and electron concentrations in the p-zone and n-zone, respectively. *n_i_* stands for the intrinsic carrier concentration in the thermal equilibrium state. Based on the depletion layer hypothesis, we obtain the charge balance condition

[3]
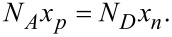


In addition, the contact potential difference of SCZ under nonzero σ can be solved as

[4]
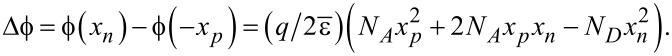


It should be noted that *D* = 0 on *x* = –*L_l_* and *x* = *L_r_* has been applied in the above.

In step II, an electric bias voltage *V* is applied on the p–n junction shown in the P-C state in Figure 1. Under the condition of low-level injection, the applied electric bias voltage is assumed to drop completely in the SCZ in conventional semiconductor physics [33]. Considering that the change of the electric field caused by NEMC under the low-level injection is very small, we still assume the applied electric bias voltage to vanish completely in the SCZ. Obviously, distributions of majority carriers and electric field are scarcely influenced by diffusion of minority carriers. The electric potential field ϕ and electric field –dϕ/d*x* are solved similarly as in step I just by replacing 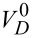 with 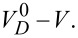 Thus, the current density from diffusion of minority carriers in p-zone and n-zone can be expressed as

[5]
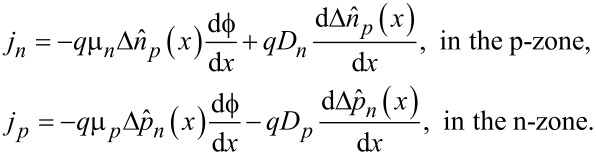


In the above, 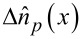 denotes NEMC in the p-zone and 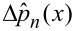 denotes NEMC in the n-zone; *j_n_* and *j_p_* are the minority current densities, respectively. (µ*_p_*, *D_p_*) and (µ*_n_*, *D_n_*) are mobility and diffusion coefficients of holes and electrons, respectively.

The continuity equation of NEMC can be obtained as follows by perturbation of small fields superposed upon step I [32]

[6]
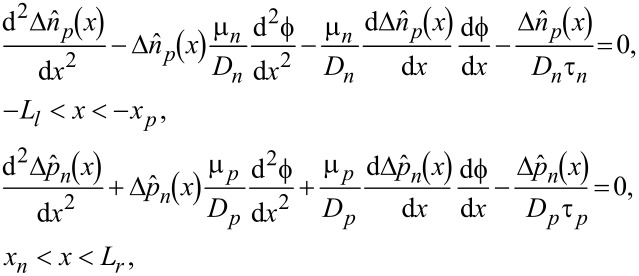


where (τ*_n_*, τ*_p_*) are the recombination lifetimes of electrons and holes, respectively. A forward-bias voltage lowers the potential barrier of the p–n junction such that majority carriers in the n- and p-zone, go across the SCZ to accumulate on the boundary at the other side as NEMC. The accumulation of minority carriers there will give rise to gradients of NEMC, which stimulates diffusion of NEMC departing from the two boundaries into the zones outside the SCZ and causes a positive current; a reverse-bias voltage increases the potential barrier such that minority carriers at the boundaries are extracted across the SCZ to form a reversal current. We note that the current across a piezoelectric p–n junction can be solved from Equation 6 together with the following boundary conditions [33]: 1) no NEMC at *x* = –*L_l_* and *x* = *L_r_*; 2) continuity of NEMC and current density at *x* = –*l_l_* and *x* = *l_r_*; 3) NEMC concentrations at the SCZ boundaries satisfy

[7]
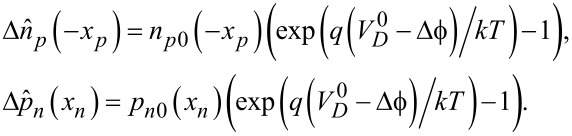


## Results and Discussion

### Quasi-electrostatic analysis of a mechanically loaded piezoelectric p–n junction under a bias voltage

Firstly, electric potential and electric field in a mechanically loaded piezoelectric p–n junction under a bias voltage are studied from a quasi-electrostatic analysis similar to step I. The bias voltage is assumed to act on the SCZ under the condition of low-level injection. The material constants of CdS rods with *c*-axis in *x*-direction are from [34–35]: *c* = 93.8 GPa, *e* = 0.44 C/m^2^, ε = 9.53ε_0_ and ε_0_ = 8.8542 × 10^−12^ F/m. The relative carrier concentrations in our calculations are taken as *p_p_*_0_ = *n_n_*_0_ = 1 × 10^21^ m^−3^ and *n_p_*_0_ = *p_n_*_0_ = 1 × 10^11^ (m^−3^), unless otherwise stated. The doping concentrations can be expressed as *N_A_* = *N_D_* = 1 × 10^21^ m^−3^. The lengths involved are taken as *L_l_* = *L_r_* = *L* = 10*l_l_*, 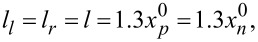 unless otherwise stated. 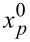 and 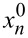 are the initial SCZ boundaries without mechanical loading or electric bias voltage. Due to the same doping, 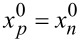 is obtained from Equation 3. They can be determined from [33] as


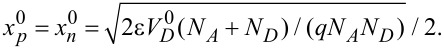


**Figure 2:**
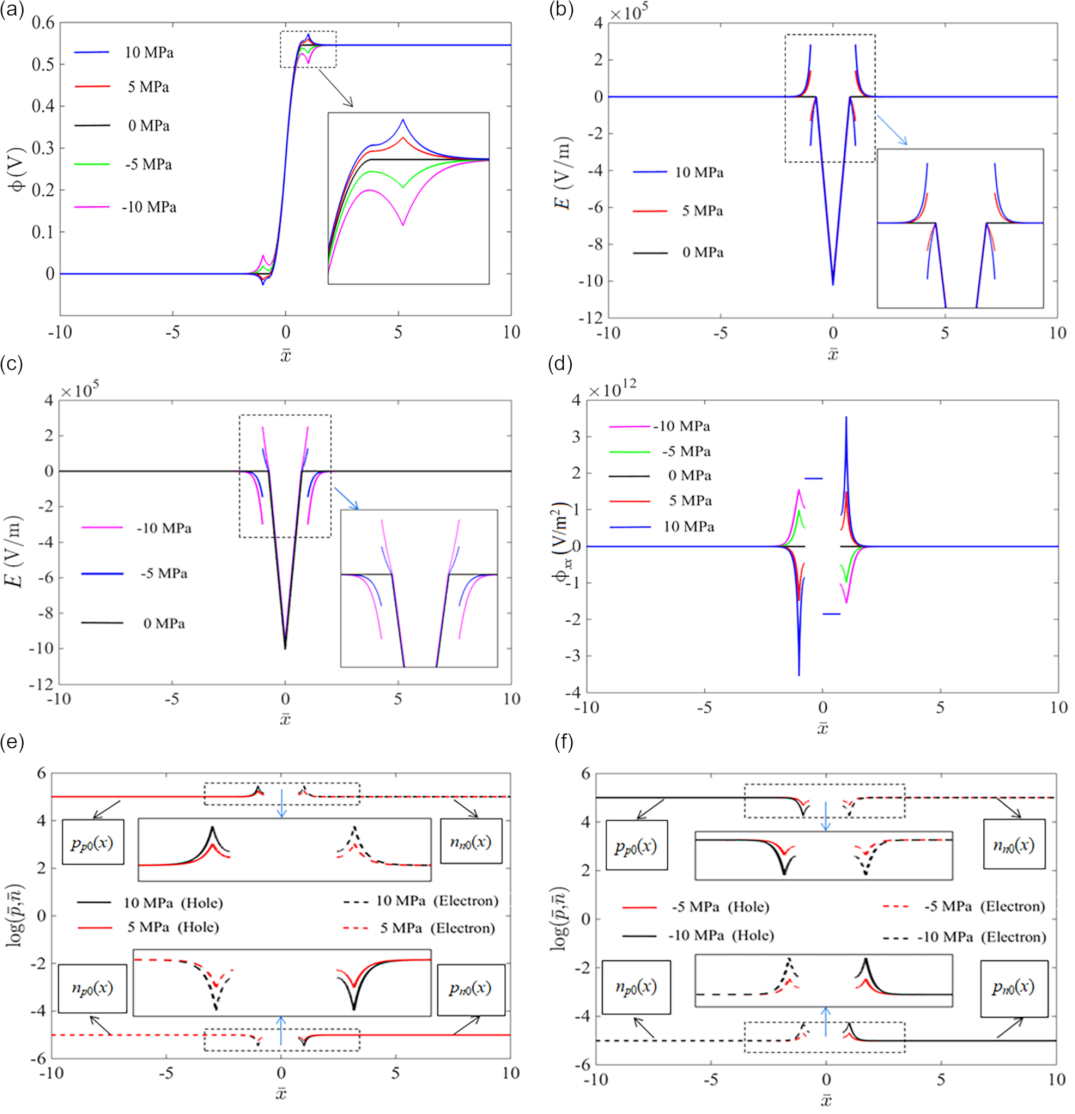
Quasi-electrostatic analysis of a mechanically loaded piezoelectric p–n junction under a bias voltage: (a) electric potential; (b) electric field under tensile stress; (c) electric field under compressive stress; (d) the second derivation of electric potential; (e) carrier concentrations under tensile stress; (f) carrier concentrations under compressive stress.

We take *V* = 0.05 V in the following quasi-electrostatic calculation: the electric potential, together with its first (related to electric field) and second derivatives, and carrier concentrations from the quasi-electrostatic analysis are shown in Figure 2 for mechanical loading, where *x̄* = *x*/*l*. Figure 2a shows a fluctuation of the electric potential induced by mechanical loading in the whole p–n junction region. Of course, a larger loading results in a stronger potential fluctuation. Moreover, the potential fluctuation mainly occurs near the loading point and peaks at *x̄* = ±1. With increasing distance from the loading points, the potential fluctuation gradually decreases due to the shielding effect of carrier redistribution on the polarized electric field. This agrees well with the results in [29]. Electric fields induced by mechanical loading are shown in Figure 2b and Figure 2c under tensile and compressive stresses, respectively. Jumps of the electric field occur at the loading points. When the piezoelectric p–n junction is exposed to a tensile stress (σ > 0), the electric field is negative (*E* < 0) between the loading points and the SCZ boundaries. The electric field induced by mechanical loading drives holes (majority carriers in the p-zone) in −1.0 < *x̄* < −*x_p_*/*l* to move left and electrons (majority carriers in the n-zone) in *x_n_*/*l* < *x̄* < 1.0 to move right. Thus, the potential barrier is increased and the SCZ size is enlarged. On the other hand, when the piezoelectric p–n junction is exposed to compressive stress (σ < 0), the electric field is positive (*E* > 0) between the loading points and the SCZ boundaries. The electric field induced by mechanical loading drives holes in −1.0 < *x̄* < −*x_p_*/*l* to move right and electrons in *x_n_*/*l* < *x̄* < 1.0 to move left. Thus, the potential barrier is lowered and the SCZ size is reduced. For convenience, the second derivation of electric potential is also shown in Figure 2d. We note from Figure 2a–d that the electric potential and its first two derivatives outside the SCZ are almost zero for σ = 0. There are no terms related to electric potential and its first two derivatives in the Shockley model [33]. However, there is a great effect of the electric field and its derivation on the diffusion of NEMC when the loading points are near the SCZ that cannot be neglected. The carrier redistribution under two different tensile stresses is shown in Figure 2e. The concentration of majority carriers increases and the concentration of minority carriers decreases with the enlargement of tensile stress. This is because the electric potential decreases (increases) in the p-zone (n-zone) under tensile loading to induce increase (decrease) the corresponding electric potential energy. A higher electric potential energy corresponds to a smaller concentration of electrons. Figure 2f shows the carrier redistribution under two different compressive stresses, with the results being opposite to that during tensile loading. Finally, it should be noted that the carrier concentrations are normalized as (*p̄*, *n̄*) = (*p*_(_*_n_*_0,_*_p_*_0)_(*x*), *n*_(_*_n_*_0,_*_p_*_0)_(*x*))/*n_i_* in Figure 2e and Figure 2f.

### Non-equilibrium analysis of a piezoelectric p–n junction under a bias voltage

The mobility coefficients of holes and electrons of CdS at 300 K are (µ*_p_*, µ*_n_*) = (0.005, 0.034) m^2^/V·s [34–35], and the corresponding diffusion constants can be obtained from the Einstein relation as (*D_p_*, *D_n_*) = (µ*_p_*, µ*_n_*)*kT*/*q*. In the following, we will focus on the influence of different mechanical loadings, different loading locations and different doping concentrations on the *I*–*V* characteristics of a piezoelectric p–n junction .

#### Effect of mechanical loading on *I*–*V* characteristics of a piezoelectric p–n junction

**Figure 3:**
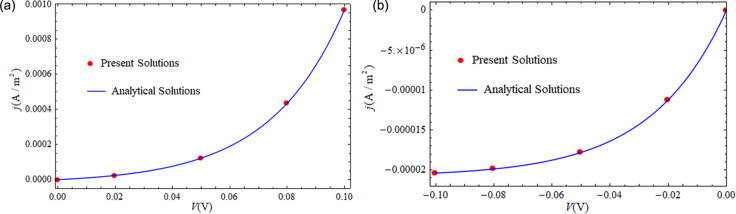
Comparison of *I*–*V* characteristics obtained from our model and the Shockley model under (a) forward-bias voltages; (b) reverse-bias voltages.

In this section, we discuss the effect of mechanical loading on the *I*–*V* characteristics of a piezoelectric p–n junction under forward- or reverse-bias voltages. Regarding the recombination lifetime of minority carriers, we take τ*_n_* = τ*_p_* = τ = 1 × 10^−9^ s to analyze the *I*–*V* characteristics of a CdS p–n junction with *N_A_* = *N_D_* = 1 × 10^21^ m^−3^. To examine the validity of our analysis model, we first calculate the *I*–*V* characteristics of the p–n junction without mechanical loading in Figure 3. The results based on the Shockley model for a pure semiconductor p–n junction is also given in the plot for comparison. Our results agree with those obtained from the Shockley model under both forward- and reversal-bias voltages. In the following, we will therefore conduct computations on the *I*–*V* characteristics using our model.

**Figure 4:**
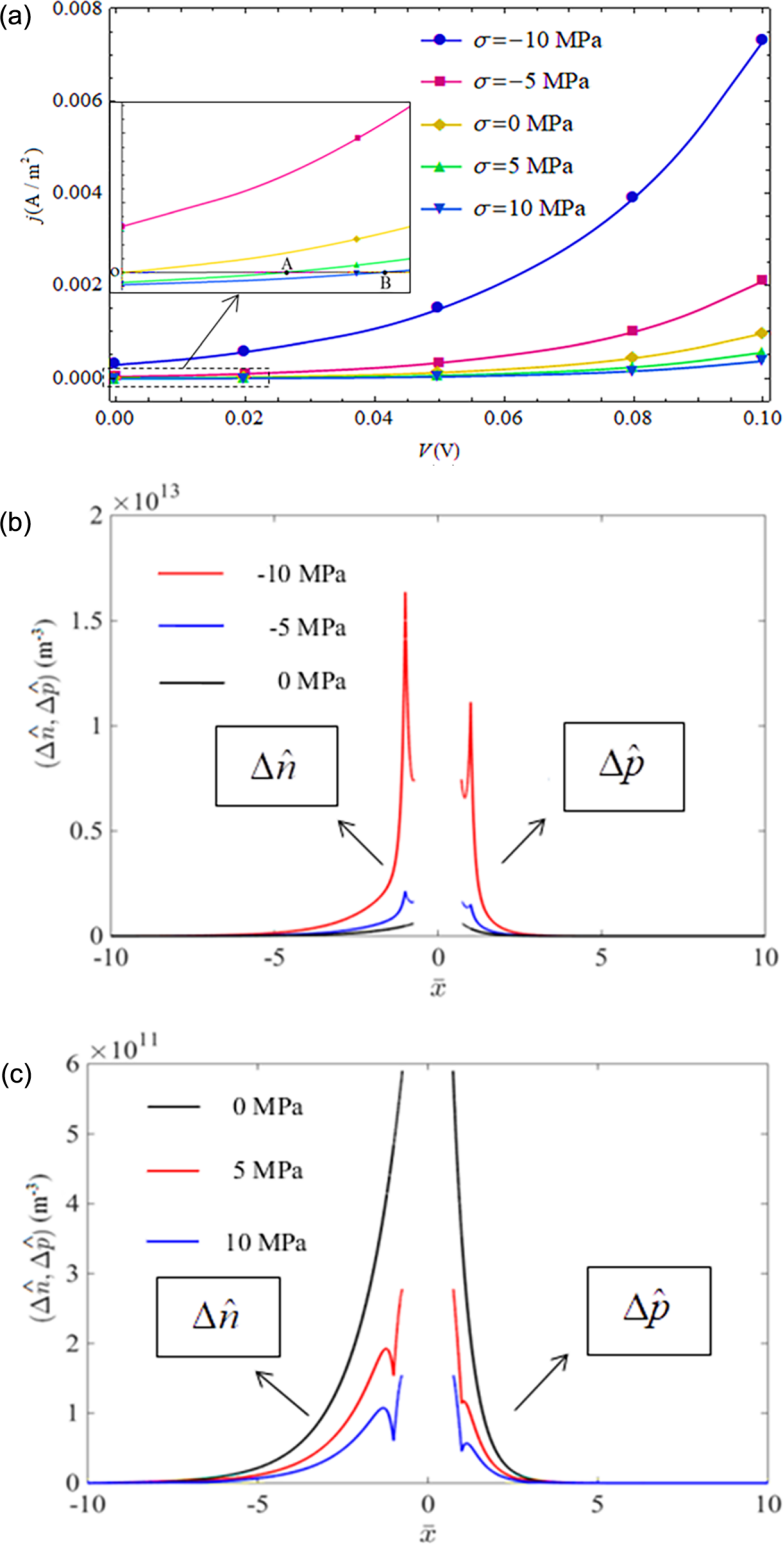
(a) *I*–*V* characteristics of a piezoelectric p–n junction under different loadings; (b) NEMC distributions at *V* = 0.05 V under different compressive loadings; (c) NEMC distributions at *V* = 0.05 V under different tensile loadings.

Figure 4a shows effect of mechanical loading on the *I*–*V* characteristics of a CdS p–n junction under forward-bias voltages for σ from −10 MPa to 10 MPa. We note that a compressive loading raises the current density and a tensile loading reduces the current density for a given forward-bias voltage. Under compressive loading, the potential barrier of the SCZ is reduced and the injected NEMC accumulate at the SCZ boundaries. The opposite happens under tensile loading. The distributions of NEMC and their accumulation at the SCZ boundaries are shown for different compressive loadings in Figure 4b and for different tensile loadings in Figure 4c, under a forward-bias voltage of *V* = 0.05 V. It follows from Figure 4 that mechanical loadings produce an obvious tuning effect on the *I*–*V* characteristics of a CdS p–n junction with piezoelectric properties. Moreover, the tuning effect described here qualitatively agrees with the result shown by Zhang et al. in [14], where a junction between a p-type non-piezoelectric and an n-type piezoelectric material was considered. In addition, we note a special phenomenon in the inset of Figure 4a that when the CdS p–n junction is under zero bias voltage, a nonzero positive current density appears under compressive loading and a negative one under tensile loading. Also, the current density vanishes at point A for σ = 5 MPa and at point B for σ = 10 MPa in the inset, while the forward-bias voltage is not zero. The above phenomenon comes from the fact that a compressive (tensile) loading reduces (raises) the potential barrier of p–n junction, equivalent to an increase (decrease) of the forward-bias voltage. This phenomenon may be applicable in force sensors.

**Figure 5:**
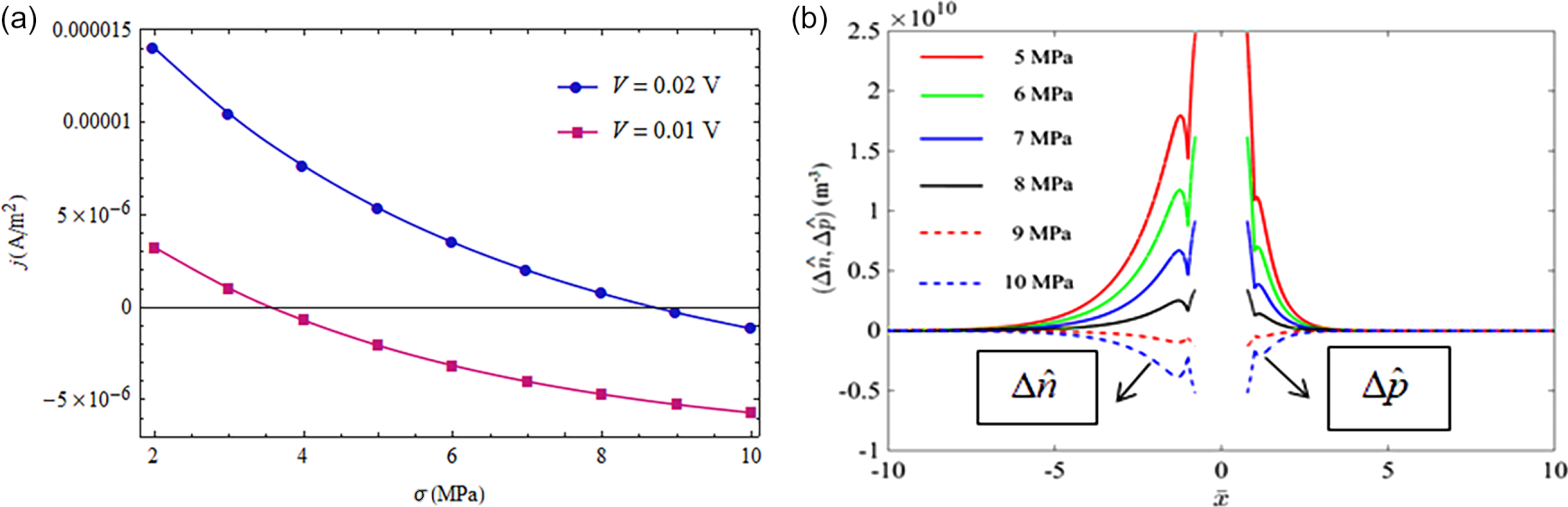
(a) Current density as a function of applied tensile stress under two constant forward-bias voltages; (b) NEMC under different tensile stresses at *V* = 0.02 V.

Figure 5 further illustrates the phenomenon of reverse current density caused by tensile loading under small forward-bias voltages. As shown in Figure 5a, the negative current density appears at a tensile loading of 3.6 MPa for a 0.01 V forward-bias voltage and at a tensile loading of 8.6 MPa for a 0.02 V forward-bias voltage. The distributions of NEMC for different tensile loadings are shown in Figure 5b with a forward-bias voltage *V* = 0.02 V. Obviously, the concentrations of minority carriers at the SCZ boundaries under a forward-bias voltage of *V* = 0.02 V for σ < 8.6 MPa are larger than the ones in thermal equilibrium, i.e., 
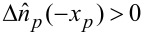
 and 
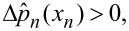
 which implies the appearance of an injection mode of NEMC. Concentrations of minority carriers at the SCZ boundaries under a forward-bias voltage of *V* = 0.02 V for σ > 8.6 MPa are smaller than the ones in thermal equilibrium, i.e., 
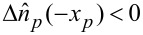
 and 
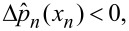
 which implies appearance of an extraction mode of NEMC.

**Figure 6:**
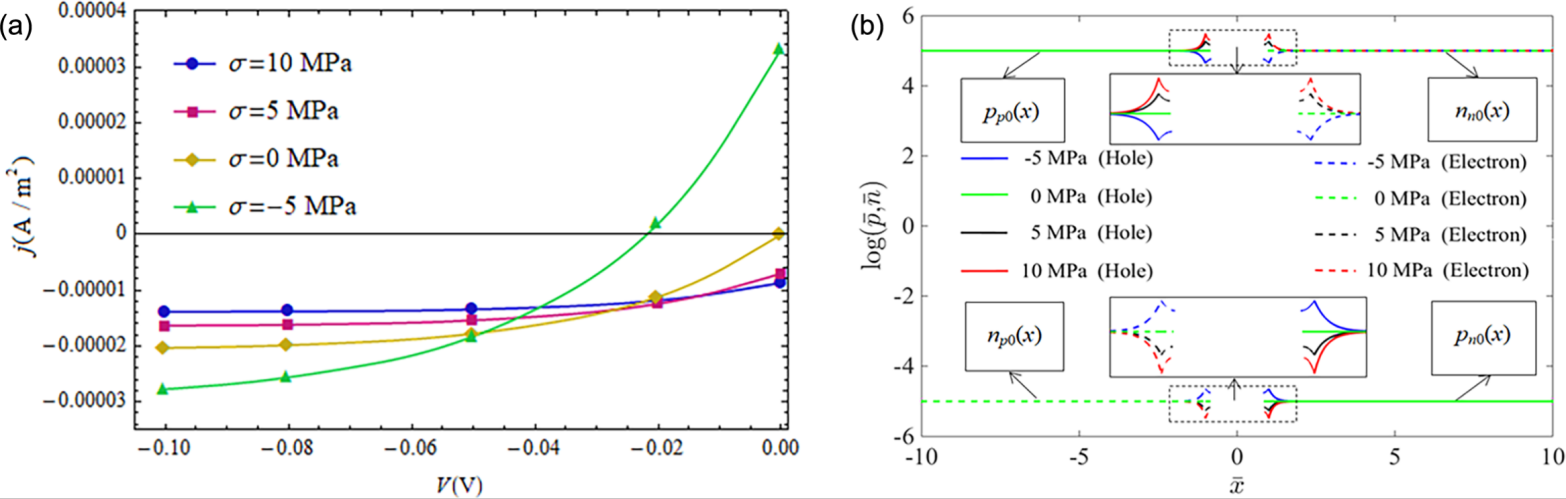
(a) *I*–*V* characteristics of a piezoelectric p–n junction under reverse-bias voltages and different applied stresses; (b) distribution of the initial carrier concentrations for *V* = −0.10 V.

Figure 6 shows the *I*–*V* characteristics of a CdS p–n junction at *V* < 0 under different values of prestress and the distributions of the initial carrier concentrations at *V* = −0.1 V. First of all, we note from the *I*–*V* characteristics of the case without mechanical loading (σ = 0) in Figure 6a that the reverse current density gradually reaches saturation –*j_s_* = *q*(*D_n_n_p_*_0_/*L_n_* + *D_p_p_n_*_0_/*L_p_*) [33] as expected for a decrease of reverse-bias voltage from 0.0 V to −0.10 V. *j_s_* becomes small (large) when the junction is exposed to tensile (compressive) loading. Compressive (tensile) stress acting on a piezoelectric p–n junction reduces (raises) the barrier of the SCZ. This means that a compressive (tensile) loading not only leads to an electric potential and an electric field in the p- and n-zone of a piezoelectric p–n junction, but also produces an equivalent forward- (reverse-) bias voltage at the SCZ. The concentrations of minority carriers (*n_p_*_0_(*x*) and *p_n_*_0_(*x*)) at the SCZ boundaries were reduced by tensile loading and raised by compressive loading, as shown in Figure 6b. Carrier concentrations in Figure 6b are normalized as (*p̄*, *n̄*) = (*p*_(_*_n_*_0,_*_p_*_0)_(*x*), *n*_(_*_n_*_0,_*_p_*_0)_(*x*))/*n_i_*. Finally, we note from Figure 6a an interesting phenomenon at *V* = 0.0 V that current density *j_s_* > 0 for a compressive loading and *j_s_* < 0 for a tensile loading, which comes from an equivalent forward- (reverse-) bias voltage on the SCZ produced by the mechanical loadings. This phenomenon may be useful in force sensors.

**Figure 7:**
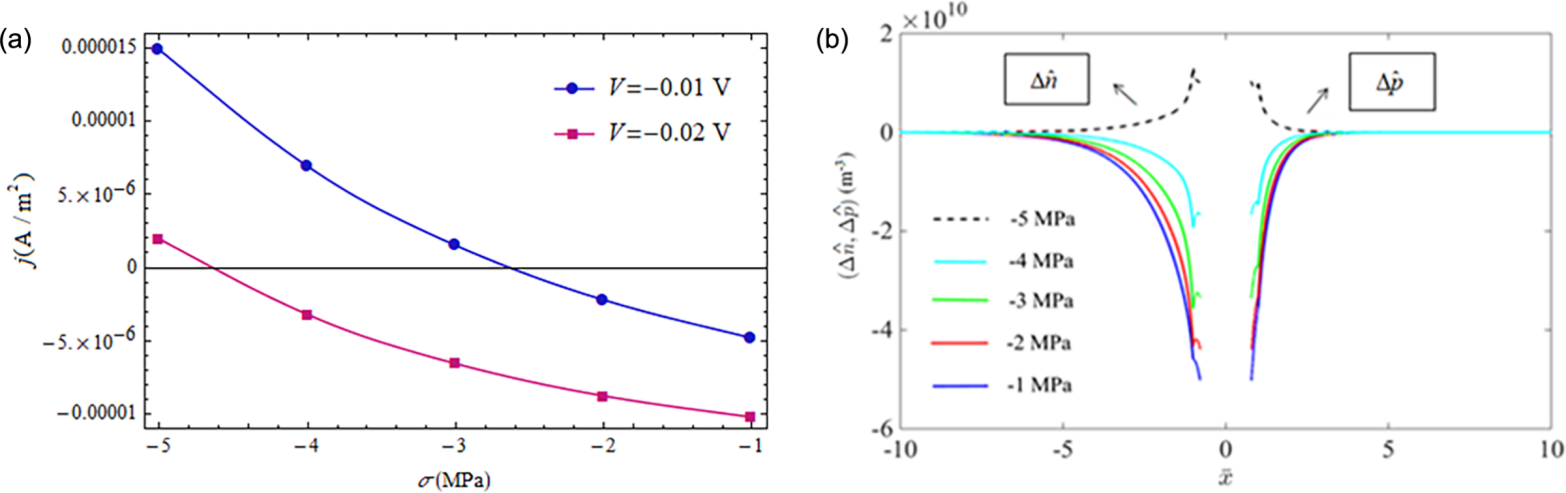
Positive current density under a reverse-bias voltage. (a) Current density under different applied compressive stresses and a fixed reverse-bias voltage; (b) NEMC under different compressive stresses at *V* = −0.02 V.

Figure 7a shows that the current density reverses from negative to positive under increasing compressive loading and a constant reverse-bias voltage. As mentioned above, a compressive loading is equivalent to a forward-bias voltage due to the piezoelectric properties of CdS. This equivalent forward-bias voltage will counteract on the reverse-bias voltage. Once the compressive stress increases to a certain value the equivalent forward-bias voltage is larger than the applied reverse-bias and the direction reversal happens for the current density of the p–n junction, for example at σ = −2.7 MPa and *V* = −0.01 V and at σ = −4.7 MPa and *V* = −0.02 V. The corresponding distribution of NEMC is shown in Figure 7b for *V* = −0.02 V. It can be seen that change of NEMC at the SCZ boundaries changes from extraction mode to injection mode when the applied compressive stress increases over −5 MPa. This direction reversal of the current density may also be of significance in force sensors.

#### Influence of loading locations on the *I*–*V* characteristics of a CdS p–n junction under forward-bias voltages

**Figure 8:**
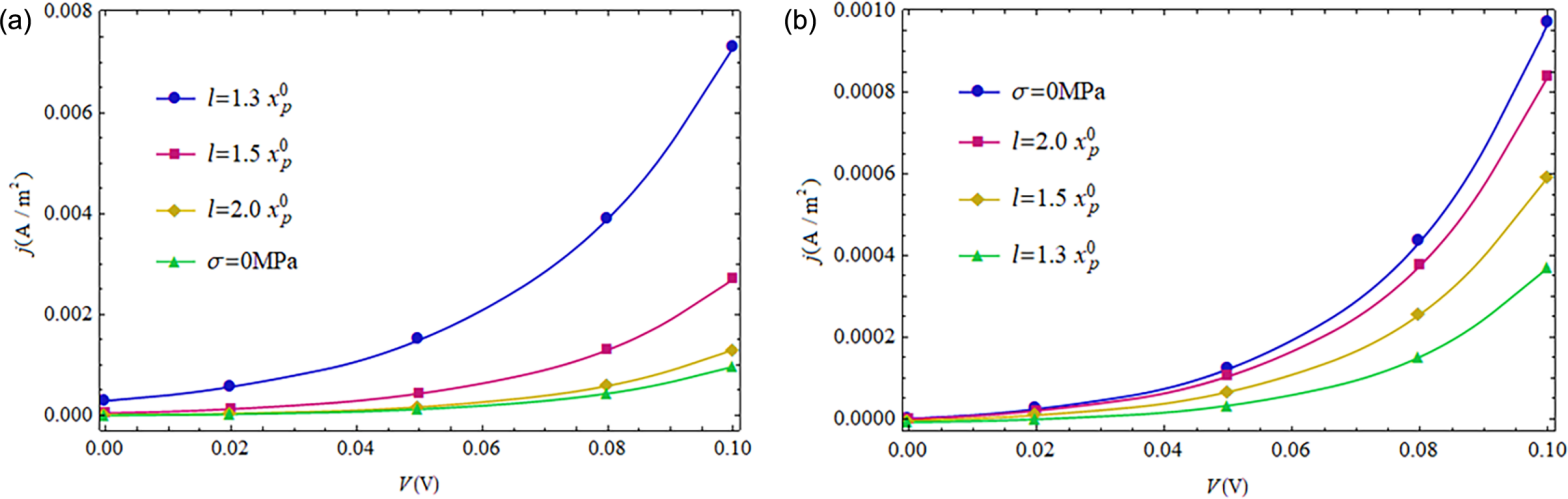
*I*–*V* characteristics for different loading locations under constant applied stress: (a) σ = −10 MPa; (b) σ = 10 MPa.

As reported in [29], the distance between the loading points and the SCZ boundaries exhibit great influence on the electrostatic quantities, that is, loading with a shorter distance produces a stronger influence on distributions of electric potential and carrier concentrations near the SCZ. Figure 8a shows this influence for compressive loading, σ = −10 MPa, and Figure 8b for tensile loading, σ = 10 MPa. It can be seen from Figure 8 that all *I*–*V* characteristics approach that without mechanical loading (σ = 0 MPa) when *l* increases from 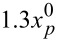 to 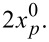 The polarization electric field is disturbed once the piezoelectric semiconductors are exposed to prestress/prestrain. Thus, the carriers will be redistributed to induce an additional electric field, which in turn has a weakening effect on the polarization electric field. Obviously, the above two electric fields will offset each other in a sufficiently large action region, i.e., at longer distances between loading point and SCZ boundary.

#### The *I*–*V* characteristics of a CdS p–n junction with different doping concentrations under forward-bias voltages

**Figure 9:**
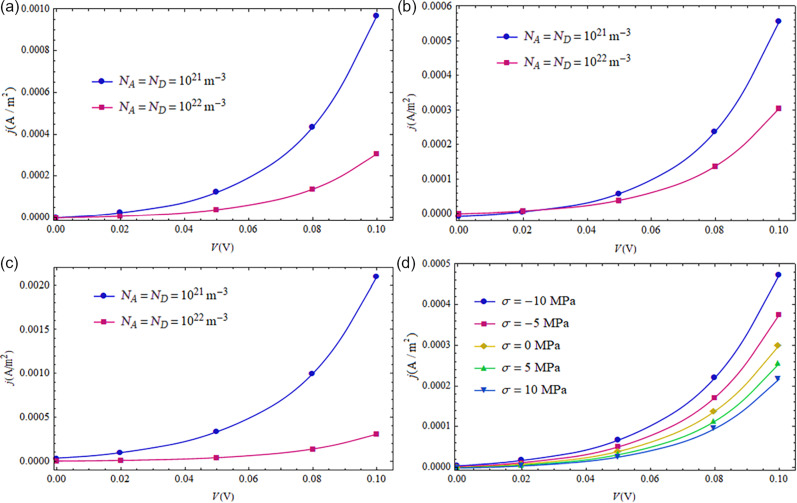
*I*–*V* characteristics of a CdS p–n junction with different doping concentrations exposed to mechanical stress: (a) σ = 0 MPa. (b) σ = 5 MPa. (c) σ = −5 MPa. (d) Effect of mechanical loadings on a CdS p–n junction with *N_A_* = *N_D_* = 1 × 10^22^ m^−3^.

In the above analysis, doping concentrations of a CdS p–n junction were kept constant at *N_A_* = *N_D_* = 1 × 10^21^ m^−3^. In this section, we study effect of the doping concentration on the *I*–*V* characteristics of a CdS p–n junction by considering another doping concentration, i.e., *N_A_* = *N_D_* = 1 × 10^22^ m^−3^. The lifetime of non-equilibrium carriers reduces with increasing doping concentrations [33]. Thus, the corresponding recombination lifetime is taken as τ*_n_* = τ*_p_* = τ = 1 × 10^−10^ s for *N_A_* = *N_D_* = 1 × 10^22^ m^−3^. The loading location is set at *l* = 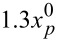 in Figure 9a–c and set at *l* = 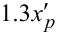 in Figure 9d, where 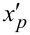 is calculated similar as above just by replacing the doping concentrations and the contact potential difference with those corresponding to *N_A_* = *N_D_* = 1 × 10^22^ (m^−3^).

The *I*–*V* characteristics of a CdS p–n junction with different doping concentrations and mechanical loadings are shown in Figure 9. Obviously, the larger doping concentrations correspond to the higher potential barrier of the SCZ. Thus, a greater current density is obtained from the lower doping concentrations (Figure 9a). We note from [21] that the semiconducting property of a piezoelectric medium leads to electric leakage, which weakens the deformation-induced electric field. Thus, the higher doping concentration yields a lower current density for a given bias voltage, as shown in Figure 9b and 9c. Figure 9d shows the mechanical tuning for a CdS p–n junction with *N_A_* = *N_D_* = 1 × 10^22^ m^−3^. A compressive loading increases the output current density while a tensile loading decreases the output current density. This phenomenon can be used to design newly piezotronic devices.

## Conclusion

We established a model to study the diffusion of NEMC and the *I*–*V* characteristics of a piezoelectric p–n junction exposed to mechanical loadings. Effects of mechanical loadings, loading locations and doping concentrations on the *I*–*V* characteristics of a CdS p–n junction under bias voltages have been investigated under the condition of low-level injection. Under tensile (compressive) loading, an equivalent reverse- (forward-) electric voltage is produced at the SCZ, and induces decreasing (increasing) current density across the CdS p–n junction under the forward-bias voltages. When a piezoelectric p–n junction is exposed to a reverse-bias voltage, the current density monotonically decreases with increasing reverse voltage at first and gradually approaches saturation. A bigger tensile (compressive) loading produces a smaller (larger) saturation current density. In addition, it has been found that the tuning effect can be enhanced by decreasing the distance between the loading points and the SCZ boundaries and by lowering the doping concentrations. The obtained results may help to design newly piezotronic devices and related experiments should be carried out in the future.
